# Applications of artificial intelligence in dementia

**DOI:** 10.1111/ggi.14709

**Published:** 2023-11-02

**Authors:** Masashi Kameyama, Yumi Umeda‐Kameyama

**Affiliations:** ^1^ AI and Theoretical Image Processing, Research Team for Neuroimaging, Tokyo Metropolitan Institute for Geriatrics and Gerontology Tokyo Japan; ^2^ Dementia Center The University of Tokyo Tokyo Japan; ^3^ Department of Geriatric Medicine Graduate School of Medicine, The University of Tokyo Tokyo Japan

**Keywords:** deep learning, dementia, machine learning, technology

## Abstract

The recent evolution of artificial intelligence (AI) can be considered life‐changing. In particular, there is great interest in emerging hot topics in AI such as image classification and natural language processing. Our world has been revolutionized by convolutional neural networks and transformer for image classification and natural language processing, respectively. Moreover, these techniques can be used in the field of dementia. We introduce some applications of AI systems for treating and diagnosing dementia, including image‐classification AI for recognizing facial features associated with dementia, image‐classification AI for classifying leukoaraiosis in MRI images, object‐detection AI for detecting microbleeding in MRI images, object‐detection AI for support care, natural language‐processing AI for detecting dementia within conversations, and natural language‐processing AI for chatbots. Such AI technologies can significantly transform the future of dementia diagnosis and treatment. **Geriatr Gerontol Int 2024; 24: 25–30**.

## Graphical abstract

We introduce the research on AI utilization in dementia, which can lead us to a better world.

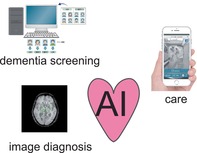

## Introduction

Research on artificial intelligence (AI) boasts a long history dating back to the early days of computers, with cycles of boom and stagnation. The rapid progress in deep learning can be attributed to the development of methods that address the vanishing gradient problem, which has impeded the training of deep neural networks and the availability of environments capable of handling big data. In addition, the use of of graphics processing units (GPUs) for parallel processing has significantly accelerated learning.

Various deep‐learning applications have been implemented in the field of medicine. For example, the AI from Google exhibited a performance comparable to that of medical experts in diagnosing retinopathy in a retinal photograph of a diabetic patient.^1^ Furthermore, Google has also developed a real‐time pathology diagnosis of cancer, which is integrated into microscopes and provides contours of cancerous tissue.^2^ Image diagnostics is one of the most advanced fields in AI within the medical domain.^3^ The authors have also conducted research on AI for distinguishing between brain blood flow single photon emission computed tomography scans in Alzheimer's disease (AD) and dementia with Lewy bodies.^4^, ^5^

Extensive efforts are also being made in the field of dementia other than in image interpretation, and there is a possibility that dementia diagnoses will undergo significant changes in the future. Here, we would like to introduce the research on AI utilization in dementia.

## Overview of recent advancements in AI

AI is a broad concept that includes machine learning, a process that allows AI to learn from many examples without requiring humans to teach the definitions. Machine learning can be broadly classified into unsupervised learning and supervised learning, with the most promising deep learning approach being the latter.

The concept of deep learning has been around for a long time; however, it is only recently that deep learning has been developed. Although a two‐layer artificial neural net was successful in the 1980s, deeper layers could not improve learning accuracy. The cause of the failure turned out to be a problem termed the vanishing gradient problem: when using the sigmoid function, the slope is at most 1/4. As a result, when stacking layers and backpropagating, the slope gradually decreases and the learning process is unable to progress. The vanishing gradient problem was overcome by changing the activation function to a rectified linear unit (ReLU) and developing new models such as ResNet. Furthermore, with advances in GPU technology on the hardware side, deep learning has advanced rapidly in recent years.

AI can perform various tasks, including image classification, object detection, natural language processing, anomaly detection, predictive analytics, and image generation. Among these, image classification and natural language processing have recently evolved.

### Image classification

Image classification is now used for face recognition, character recognition, and autonomous driving technology. The advent of convolutional neural networks (CNNs) has significantly improved image classification performance. In particular, since the University of Toronto's Alexnet^6^ won the 2012 ImageNet Large‐Scale Visual Recognition Challenge (ILSVRC), using deep learning for image recognition has rapidly gained popularity. The error rate has now been reduced to less than 5%, surpassing human recognition capabilities.

**Figure 1:**
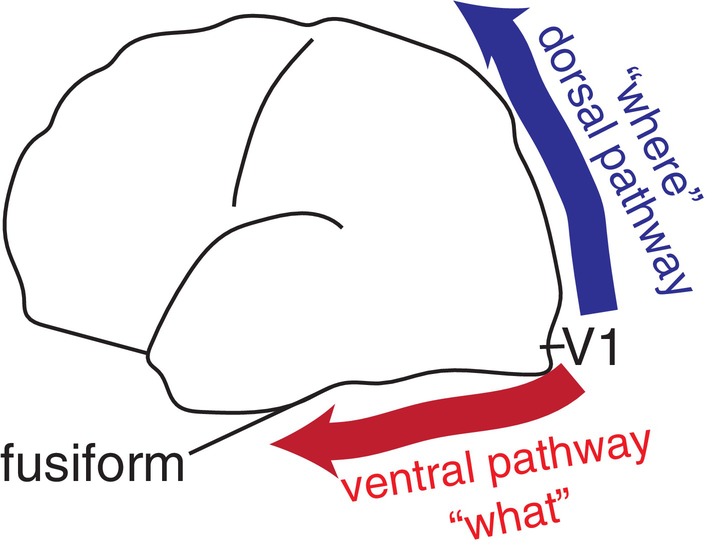
Dorsal and ventral visual pathways in humans.

A CNN works by repeatedly passing various filters over images, which strongly resembles the neural visual information processing of the brain in the primary visual cortex (V1).^7^, ^8^, ^9^ This is a compelling example of using computers to model the brain and gain a deeper understanding of its functions. Of the two visual pathways (dorsal and ventral) (Fig. 1), AI classifies images in a similar way to image processing in the ventral pathway. Object‐selective neurons exist in the inferotemporal cortex of primates^10^ or the human fusiform gyrus.

### Natural language processing

Natural language processing (NLP) has recently gained significant attention. As such, voice assistants such as Siri® (Apple), Google Assistant® (Google), and Alexa® (Amazon) have become familiar companions. Moreover, the performances of AI‐based translation, speech recognition, chatbots, and other NLP applications have significantly improved.

In NLP, enabling computers to handle language effectively is the initial challenge. Although character encoding has been in operation, it merely assigns labels to characters without representing their meaning. Colors are represented in a 3‐dimensional space corresponding to three cones. The sense of smell is expressed through combinations of 396 G‐protein coupled odorant receptors in humans. Similarly, in 2013, the word2vec technique was introduced, in which the meaning of words was represented using several hundred‐dimensional vectors. This technology advantageously brings similar meanings closer together and allows for operations such as “king – queen = man – woman”. In addition, the proximity of the two words can be determined by computing the inner products of the two vectors. With this word vector representation, computers have gained the ability to handle the semantics of natural language, marking a significant advancement in NLP. Furthermore, based on the word2vec vectorization technique, a technology termed doc2vec allows the representation of entire documents as vectors, similar to how words are vectorized.

Models such as recurrent neural networks (RNNs) have long been used to progress through a sequence of words using a neural network. Given that word order is important in language, sequential processing reads one word at a time from the beginning of a sentence. However, the drawbacks to this approach, including the difficulties of processing long sentences accurately, vulnerability to the vanishing gradient problem, and challenges in parallel processing, make it difficult to scale up the models. Modified versions such as Long Short‐Term Memory (LSTM) and Gated Recurrent Unit (GRU) were developed to partially overcome RNNs' issue of quickly forgetting information.

Transitioning from sequential to parallel processing allows the creation of “Large Language Models (LLMs)”. The introduction of the “transformer”, incorporating “attention” mechanisms,^11^ allowed NLP AI to break free from the limitations of RNNs. Although word order is important in NLP, positional information can be overcome by separate encoding.

Models using transformers, such as Bidirectional Encoder Representations from Transformers (BERT; Google) and Generative Pre‐trained Transformer‐3 (GPT‐3; OpenAI) have undertaken the current world of NLP with the capability of parallel processing. These models were pre‐trained on vast information from almost the entire Internet. The effectiveness of LLMs can be understood by examining ChatGPT.

## Applications in the field of dementia

### Facial dementia screening: Image classification

We are currently investigating a method to diagnose dementia using facial photographs.^12^ Many people question whether dementia can be diagnosed based on facial features. Aging is a comprehensive process affecting the entire body. Facial assessment of perceived age, which is how old a person appears based on their facial features, is known to correlate with lifespan, telomere length,^13^ arteriosclerosis,^14^ and osteoporosis.^15^ It serves as a biomarker of aging. Dementia is also a process of aging; thus, we believe that it has an impact on appearance. Therefore, we demonstrated that perceived age is significantly more strongly correlated with cognitive function (Mini‐Mental State Examination [MMSE], Vitality Index) than with chronological age.^16^ This suggests that facial appearance is affected by cognitive function. Advanced dementia tends to manifest as a distinctive facial appearance, which is hardly noticeable at the level of mild cognitive impairment (MCI).

**Figure 2:**
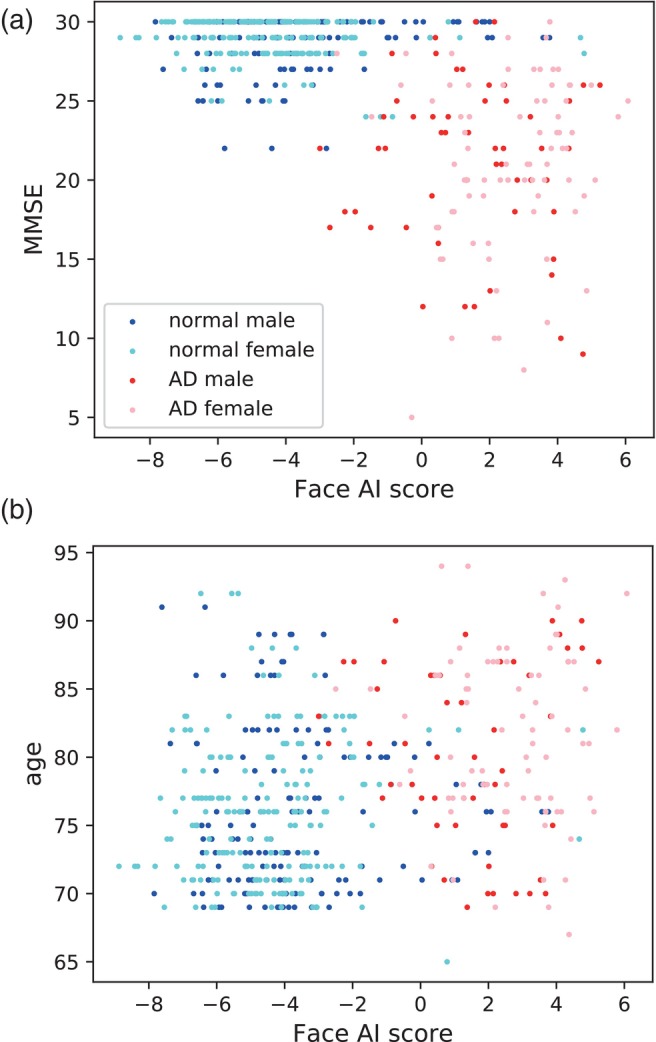
Correlation between Face AI score and (a) MMSE, (b) chronological age. Face AI score exhibited a strong correlation with MMSE score (a: r=−0.599, t=−16.40, $p=2.47\times 10^{-48}$). It showed a relatively weak correlation with age (b: r=0.321, t=7.44, $p=4.57\times 10^{-13}$). The difference in correlation coefficients was significant ($p=3.25\times 10^{-35}$).

Hence, we attempted a classification task using deep learning to distinguish between photographs of patients with AD and those of normal individuals. Although some models failed to learn, the relatively new Xception model achieved an impressive accuracy of 92.56% and an area under the curve (AUC) of 0.9717. Moreover, we calculated a value called the Face AI score before applying the sigmoid function to the final layer. The correlation between Face AI score and MMSE (r=−0.599) was significantly stronger than that between this score and chronological age (r=0.321), with a p‐value of $3.25\times 10^{-35}$ (Fig. 2).

Even among a population with many patients with MCI, the fact that AI achieved discrimination that is hardly perceptible to the human eye is remarkable.

Expectations are high for early dementia detection through facial recognition, as it could offer a non‐invasive, time‐efficient, and cost‐effective screening method. To extend this study, more facial photographs need to be collected. We collected photographs of older drivers who came for cognitive assessments at the driver's license examination center in collaboration with the National Police Agency and the Metropolitan Police Department. We are planning a multi‐institutional study to collect data with support from the Japan Agency for Medical Research and Development (AMED) to advance this research.

### Fazekas classification: Image classification

Leukoaraiosis (white matter hyperintensities) sometimes requires classification. Thus, we are currently studying AI for the Fazekas classification, a classification for leukoaraiosis.

In 2020, the Tokyo Metropolitan Institute for Geriatrics and Gerontology received a budget from the Tokyo Metropolitan Government for the establishment of the Integrated Research Initiative for Living Well with Dementia (IRIDE). One of the key pillars of this institute is the AI diagnostic system.

The Fazekas project was a collaboration with the Matsuo Lab in the Graduate School of Engineering, the University of Tokyo. Preliminary results showed reasonably good discrimination; however, we are currently making efforts to achieve even better performance.

### Microbleed detection: Object detection

Microbleeds are commonly observed in cerebral amyloid angiopathy, and it has been suggested that they are associated with AD. Additionally, microbleeds are a major risk factor for dementia.^17^ Furthermore, when antibody therapy targeting amyloid is used as a disease‐modifying therapy, there is a significant likelihood of microbleeds, known as amyloid‐related imaging abnormalities (ARIAs).

**Figure 3:**
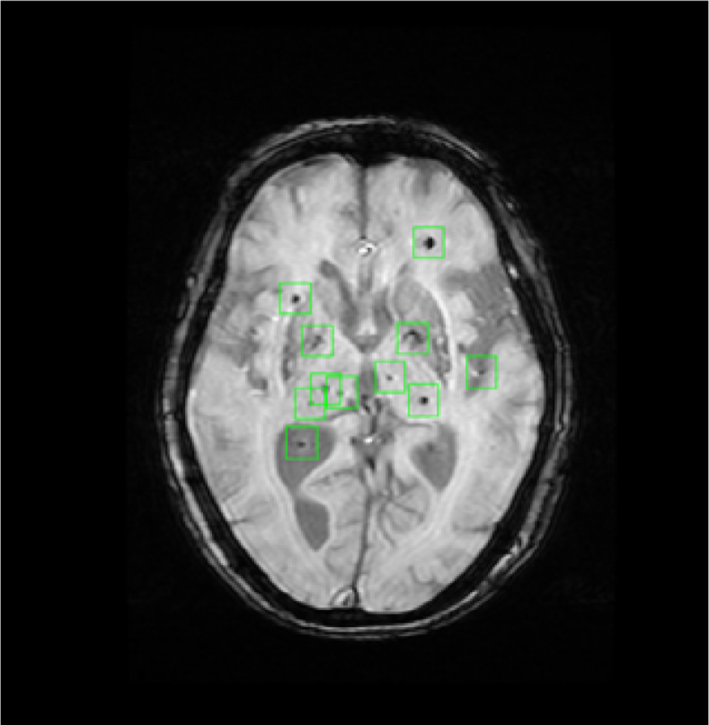
Microbleeds detected by AI.

This microbleed‐detection AI project is also a collaboration with the Matsuo Lab in the Graduate School of Engineering, University of Tokyo. We used the “You Look Only Once” (YOLO)‐based model. Microbleeds were detected, as shown in Figure 3. We hope that this microbleed‐detection program will be beneficial in clinical settings in the future.

### Care support: Object detection

Caring for individuals with dementia is a significant challenge. Although most AI research in the dementia field focuses on diagnosis, the future landscape of dementia care could be transformed by AI technologies. One notable project in this direction is HitomeQ® by KONICA MINOLTA, a comprehensive care support system. We are currently engaged in a collaborative endeavor with KONICA MINOLTA, supported by AMED.

**Figure 4:**
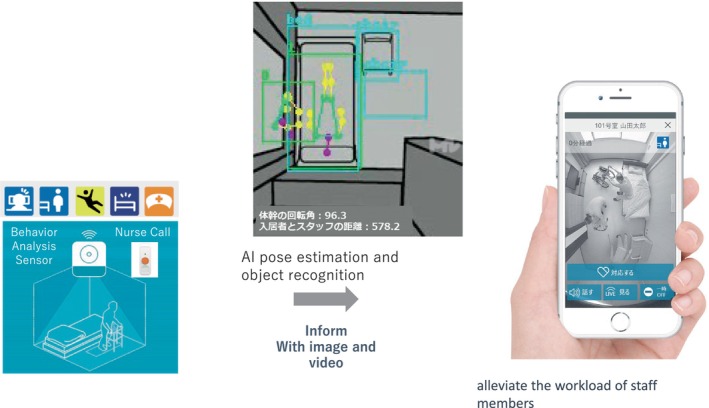
Overview of HitomeQ. The posture of the residents was estimated from a camera on the ceiling using AI. By detecting and notifying events such as falls, the burden on nursing care staff is reduced.

HitomeQ® serves as an integrated care support system that monitors user activities to alleviate the workload of caregiving staff (Fig. 4). To track residents, HitomeQ employs AI‐based pose estimation, enabled by millions of meticulously annotated training images developed by KONICA MINOLTA. This aspect distinguishes their approach, making it difficult to replicate.

Personalized care services grounded in accurately assessing the current condition and residual capabilities of older people are pivotal. The philosophy of the long‐term care insurance system emphasizes “preserving dignity and promoting self‐reliance.” However, the evaluation required for personalized care often relies on an observer's expertise and is time‐ and context‐limited. Therefore, an objective and continuous assessment method is required. Furthermore, the COVID‐19 crisis has brought about new challenges, with reduced social interaction leading to declining motor and cognitive functions in the older population. Consequently, assessing these functions while minimizing contact and providing tailored care and rehabilitation is crucial.

Our collaborative research integrates data from multi‐sensor technologies and care records shared by caregivers. AI analysis of these data facilitates objective and regular assessments of motor and cognitive functions without the conscious involvement of older people. This approach aids in the development of technology for the automatic updating of care and rehabilitation plans. Our main research goals are as follows.

- Develop a platform to integrate and manage diverse sensing devices.
- Measure motor and cognitive functions using various sensing data (e.g., behavior, sleep, conversation, facial expressions, and interactions in living and communal areas).
- Create and automatically update care and rehabilitation plans based on measured functions and shared care records.
- Validate the approach in care facilities by comparing measured functions and care plans.

The utilization of AI technologies and quantitative assessment through sensing technology aims to enhance caregivers' motivation, reduce their burden by making the effects of care visible, and streamline care and rehabilitation plans. Simultaneously, this approach efficiently maintains the residual functions of older individuals and reduces strain for families and care facilities. Ultimately, it holds the promise of boosting care productivity and promoting self‐reliance among older people, contributing to the optimization of long‐term care insurance benefits.

### Dementia screening with conversation: Natural language processing

FRONTEO has completed clinical trials for a dementia detection system using conversations. Thus, it is anticipated that this system will soon be introduced in clinical practice.

FRONTEO's AI engine, KIBIT®, has been developed as a vectorization system, like word2vec and doc2vec. Analytically solving the inner product calculation of word and sentence vectors allows the provision of insights into the topics covered in the conversation. This process leads to dementia classification using deep learning based on a matrix of inner products between words and sentences.

KIBIT® was more effective than bioBERT^18^ in extracting the literature necessary for systematic review.^19^ Compared with other models such as transformers, KIBIT® is relatively simple and lightweight, yet it seems to construct vectors with sufficient information.

Innovative efforts have been made to go beyond traditional text analysis methods, utilizing not only the original text but also its counterpart in parts of speech. Rather than focusing solely on the content of the text, these methods delve into linguistic features, such as word usage and syntax. Computers do not need to understand text content. Thus, this approach aims to capture the linguistic peculiarities associated with cognitive decline, including changes in speech patterns and grammatical structures.

In an initial study conducted at the Department of Psychiatry, Keio University, the system exhibited a 90.0% accuracy rate in discriminating between healthy individuals, those with MCI, and those with dementia.^20^ Currently, the system focuses on distinguishing healthy individuals/those with MCI from those with dementia from a medical device perspective. This suggests that the potential of the system goes beyond dementia detection and may extend to the detection of MCI as well.

While the system's success at Keio University Hospital in Tokyo provides promising results, further investigation is necessary to determine its effectiveness across different dialects and languages. A nationwide clinical trial involving multiple medical institutions should address this issue.

The use of language‐based AI technology in medical devices is a global phenomenon. Once formally approved, this AI‐based dementia diagnosis support program will be the world's first of its kind. While AI devices in Japan might be trailing behind those in Europe and the United States, pioneering endeavors such as this are undeniably positive. Moreover, language barriers inherent in AI technologies suggest that foreign manufacturers may find it challenging to enter the Japanese market. Conversely, Japanese manufacturers could potentially enter markets with large populations, such as in English, Chinese, and Spanish‐speaking regions.

### Chatbot: Natural language processing

IRIDE is developing a chatbot for emotional support for older people, in collaboration with MindShift.

Initially, MindShift was fixed to its proprietary inference engine. However, one of the authors of this paper strongly argued that, in the current era, it would be outdated if transformers were not adopted. Eventually, our team with MindShift introduced GPT‐2. Consequently, a well‐functioning system is gradually being developed.

## Bonus

Finally, we include bonus performances as a form of entertainment. A generative adversarial network (GAN) is an AI that generates or transforms images,^21^ which can be used for tasks such as improving the quality of medical images.^3^, ^22^ A cycle GAN is a type of GAN that can change a horse to a zebra, for example. We created a program using a cycle GAN that converts cognitive decline to normal and vice versa.

**Figure 5:**
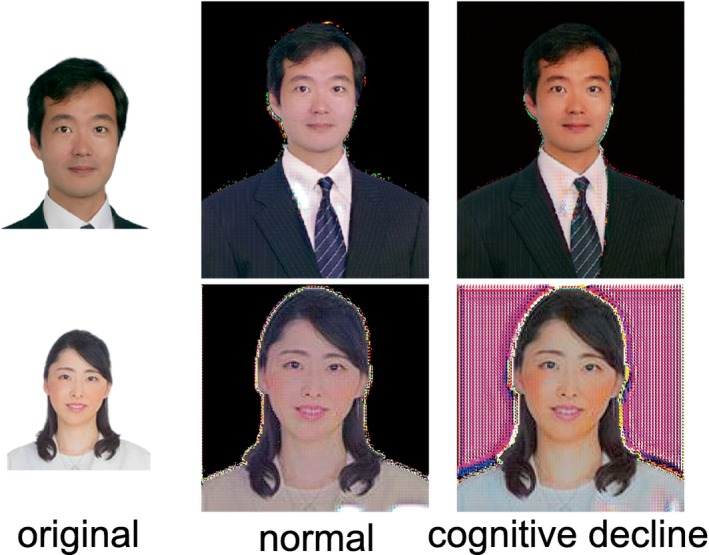
Cycle GAN that changes normal faces to cognitively impaired faces and vice versa. The left column shows the authors' original faces. The middle column shows faces that were converted to normal faces from the original ones, and the right column shows faces that were converted to cognitively impaired faces from the originals.

A cycle GAN was used to swap faces between normal and cognitively impaired subjects (Fig. 5). Both converted faces are visually indistinguishable. However, when face image was transformed into cognitive impairment, there seems to be a slightly more noticeable presence around the nasolabial folds.

## Conclusion

CNNs have advanced image classification. Additionally, NLP has seen explosive development with the emergence of transformers.

Various attempts have been made to treat dementia using AI. Dementia discrimination from facial photographs, image diagnosis using MRI, care robots, dementia discrimination from conversations, and chatbots are expected to help the growing number of people with dementia. Thus, the future of dementia diagnosis and treatment can be significantly transformed using AI technologies.

## Disclosure statement

The authors declare no conflict of interest.

## Data Availability

Data sharing is not applicable to this article as no new data were created or analyzed in this study.references.
